# Increased Systemic Th17 Cytokines Are Associated with Diastolic Dysfunction in Children and Adolescents with Diabetic Ketoacidosis

**DOI:** 10.1371/journal.pone.0071905

**Published:** 2013-08-27

**Authors:** William H. Hoffman, Gregory G. Passmore, David W. Hannon, Monica V. Talor, Pam Fox, Catherine Brailer, Dynita Haislip, Cynthia Keel, Glenn Harris, Noel R. Rose, Irma Fiordalisi, Daniela Čiháková

**Affiliations:** 1 Section of Pediatric Endocrinology, Georgia Regents University (formerly Georgia Health Sciences University), Augusta, Georgia, United States of America; 2 Medical Laboratory, Imaging, and Radiologic Sciences, Georgia Regents University, Augusta, Georgia, United States of America; 3 Section of Pediatric Cardiology, The Brody School Of Medicine, East Carolina University, Greenville, North Carolina, United States of America; 4 Department of Pathology, The Johns Hopkins University School of Medicine, Baltimore, Maryland, United States of America; 5 Section of Pediatric Critical Care, The Brody School of Medicine, East Carolina University, Greenville, North Carolina, United States of America; 6 Section of Pediatric Endocrinology, The Brody School of Medicine, East Carolina University, Greenville, North Carolina, United States of America; 7 The W. Harry Feinstone Department of Molecular Microbiology & Immunology, The Johns Hopkins University Bloomberg School of Public Health, Baltimore, Maryland, United States of America; New York University, United States of America

## Abstract

Diastolic dysfunction suggestive of diabetic cardiomyopathy is established in children with T1DM, but its pathogenesis is not well understood. We studied the relationships of systemic inflammatory cytokines/chemokines and cardiac function in 17 children with T1DM during and after correction of diabetic ketoacidosis (DKA). Twenty seven of the 39 measured cytokines/chemokines were elevated at 6–12 hours into treatment of DKA compared to values after DKA resolution. Eight patients displayed at least one parameter of diastolic abnormality (DA) during acute DKA. Significant associations were present between nine of the cytokine/chemokine levels and the DA over time. Interestingly, four of these nine interactive cytokines (GM-CSF, G-CSF, IL-12p40, IL-17) are associated with a Th17 mediated cell response. Both the DA and CCL7 and IL-12p40, had independent associations with African American patients. Thus, we report occurrence of a systemic inflammatory response and the presence of cardiac diastolic dysfunction in a subset of young T1DM patients during acute DKA.

## Introduction

Since being described four decades ago, diabetic cardiomyopathy (DCM) [1] has come to be recognized as an independent phenotype of diabetic cardiac disease. DCM is characterized by an abnormal myocardial performance unrelated to coronary atherosclerosis or hypertension [2]–[4]. There is convincing echocardiogram (ECHO) evidence of diastolic dysfunction in a significant number of children, adolescents [5]–[8] and young adults with T1DM [9]–[12]. These studies suggest that DCM, a major cause of heart failure, has an early onset in some patients with T1DM. A positive correlation has been reported between stable suboptimal metabolic control and diastolic dysfunction [7], [8], [13]; however this has not been a consistent finding [6], [14], [15].

Insulin deficiency in T1DM results in a complex metabolic stress, including: hyperglycemia [16]; hyperlipidemia [17]; ketonemia [18]; and variable intermittent insulin resistance [19], each being a metabolic risk factor in the pathogenesis of DCM. Altered insulin signaling also perturbates cardiac metabolism, with augmentation of free fatty acid (FFA) utilization and decrease in glucose consumption [20]. The metabolic instability in T1DM leads to increased oxidative stress [21] and the oxidation of various metabolites. Oxidation products such as oxidized lipoproteins interact with innate immune receptors [22] causing a low-grade systemic inflammation. Type 1 diabetes is marked by an increase of inflammatory cytokines/chemokines, such as IL-6; sCD40L [23], [24]; IL-8 [25]; IL-1α; IL-2; IL-4; IL-5; IL-10; granulocyte-macrophage colony-stimulating factor (GM-CSF); macrophage inflammatory protein (MIP)-1α; MIP-1β; and activation of normal T cell expressed and secreted (RANTES) [24].

In this study we examine diabetic ketoacidosis (DKA) and the occurrence of systemic inflammatory response (SIR) and the presence of cardiac diastolic dysfunction. We have shown previously that DKA and its treatment accentuates the systemic immune inflammatory cytokines IL-1β, IL-6, TNF- α and IL-8 in patients. In addition, we found an increased level of regulatory IL-10 prior to treatment. With the initiation of treatment the inflammatory cytokines increased and IL-10 decreased [26]. These findings were recently confirmed by Karavanaki and colleagues [27]. In addition to the increase of inflammatory cytokines, the inflammatory state of acute DKA is shown by findings of complement active peptides [28]; acute phase proteins [29], [30]; and T-lymphocyte activation [31], [32]. We hypothesized that the acute SIR during severe DKA and its treatment is a cause of acute myocardial diastolic dysfunction. Reasons to study the association of an immune insult on the myocardium in young patients with DKA are: 1) to avoid the confounding interaction of the chronic vascular complications of long-term T1DM; 2) to increase the understanding of inflammatory cytokines in the pathogenesis of clinical immune myocarditis/cardiomyopathy [33], [34]; and 3) to provide insight for timely intervention into the morbidity and mortality of cardiovascular complications of T1DM [35]. We addressed this question by assaying an array of systemic cytokines/chemokines and performing echocardiograms (ECHO) during and after correction of severe DKA using an established DKA treatment protocol [36], [37].

## Materials and Methods

### 1. Study Sample

A total of twenty-two children and adolescents between the ages of 9.5 and 17 years, presenting with diabetic ketoacidosis and total CO_2_ = /<12 mmol/L were enrolled. The study was approved by the IRB at East Carolina University Brody School of Medicine. Informed consent was signed by the legal guardian and assent from the patients over 7 years when not prohibited by severity of illness. In such cases, patient assent was obtained when clinical improvement permitted. Patients referred from outlying hospitals were stabilized prior to transport after consultation with the accepting attending physician in the Pediatric Intensive Care Unit. Patients were managed according to previously published guidelines [36], [37]. Pretreatment values were obtained for blood pressure (BP), heart rate (HR), complete blood count (CBC), glucose, electrolytes, urea nitrogen (BUN) and creatinine (Cr) at the referring hospital. The start of therapy was defined as the initiation of continuous intravenous insulin. In addition to a pretreatment blood pressure (BP), BPs were recorded hourly based on measurements with an automated oscillometric device and appropriately sized BP cuff. BP determinations were available for 19 time periods including (6–12 hrs, during DKA treatment) (T1), discharge (48 hrs), baseline ECHO (2–4 wks post discharge) (T2) and baseline cytokines/chemokines (at 3 mons) (T3). Blood glucose was obtained hourly and electrolytes, BUN and Cr were measured every two to four hours. A repeat CBC with differential was repeated at 24 hrs. None of the patients were known to have hypertension, diabetic retinopathy, nephropathy or coronary artery disease. Exclusion criteria were a history or physical findings suggestive of an acute or chronic infection, emotional or physical disability or autoimmune conditions other than chronic lymphocytic thyroiditis.

### 2. Echocardiograms

Standard two dimensional echocardiograms with Doppler velocity flow were obtained at times T1 (6–12 hrs, during DKA treatment) and T2 (2–4 wks post discharge, baseline ECHO). Patients were studied in a 45 degree sitting position. Standard two dimensional (2D) views were obtained and ventricular ejection fractions were calculated from the two dimensional images using a standard volume calculation package. Doppler flow profiles were measured just distal to the tips of the valve leaflets for peak velocities and mitral E to A filling ratio, mitral deceleration time and mitral valve isovolumetric relaxation time by standard technique and averaged from the three best Doppler profiles to study left ventricular filling.

Comparisons were made for ECHO derived measures of systolic and diastolic performance at times T1 versus T2 (baseline ECHO) for all 17 patients in aggregate. The individual patient echocardiographic measures at T1 were compared to individual measures at T2 to determine if any subset of patients had a systolic or diastolic abnormality (T1). The echocardiograms and calculations were performed by one of the authors (DH), who was masked with regard to the cytokine results.

### 3. Cytokines/chemokines and Troponin I

The first blood sample for cytokine/chemokine assay (T1) was obtained between 6–12 hrs after the start of treatment. This time was chosen as T1 since the logistics of transfer from another hospital precluded obtaining an earlier sample, and this time has been used for sample collection as the second time point in previous studies of DKA [26], [38], [39]. Subsequent samples were obtained at 2–4 wks (T2) and at 3 mon post discharge (baseline cytokine) (T3). All samples were obtained from freely flowing access sites, immediately placed in chilled EDTA tubes and immediately centrifuged at 4°C at 2,000 RPM for 20 min. The plasma was separated and stored at −80°C until assayed. Troponin I was obtained at 12 and 24 hrs.

The cytokines and chemokines were measured using Millipore Map detection kit (Austin, TX), based on the Luminex xMAP technology, that employs the use of fluorescent coated beads coated with capture antibody. The plasma samples were thawed at room temperature. A volume of 25 microliters was mixed with the beads, incubated overnight, washed and then detected with the use of a biotinylated detection antibody. The reaction mixture was incubated with Strepavidin-PE conjugate to complete the reaction on the surface of the microbeads. The microbeads were then passed through a laser which excites the internal dye and a second laser excites the PE fluorescent dye. The processor identifies each microbead and quantifies the result of the bioassay based on the fluorescent reporter signals. The outcome of the assay was read on the Bio-Plex 200 system from Bio-Rad using the Bio-Plex Manager 6.0 software. Troponin I was assayed using the Siemens Centaur Tn1-Ultra assay (Deerfield, IL).

### 4. Statistical Analysis

The patients' demographic characteristics are described as group mean +/− standard deviation or as median values with data range. Associations between demographic characteristics were tested with Fisher's Exact test or Chi-square. Blood chemistries and cytokines/chemokines are represented as group means +/− standard deviations. Correlation and regression analyses were used to determine the strength of relationships between cytokines/chemokines and blood chemistries, systolic(S) and diastolic(D) BPs, as well as cytokines/chemokines and ECHO values. Longitudinal differences between blood chemistries, BPs, and ECHO variables were tested with repeated measures ANOVA and T-tests. Comparable non-parametric analyses were utilized when data sets did not meet parametric criteria. Two-factor repeated measures ANOVA were used to investigate interactions between ECHO and cytokine/chemokine variables. NCSS 8 Statistical Software (http://www.ncss.com) was used for the statistical analyses.

## Results

### 1. Patient Demographics

Four of the 22 patients were dropped from the study for various non-medical reasons, and one was dropped because of pancreatitis. The study involved 17 patients with DKA and an average age of 13.76 yrs (range: 9.7–16.9). The mean (SD) duration of T1DM for the 11 previously diagnosed patients is 6.36 (4.02) yrs. Six patients were newly diagnosed at the time of admission. The mean (SD) T1DM duration for all 17 patients was 4.12 (4.29) yrs. There were 10 females and 7 males, 6 Caucasian (C) and 11 African American (AA) (Table 1). Sixteen of the patients were stabilized at a referring hospital after consultation with staff at East Carolina Medical Center, where the patients were then transferred to the PICU for treatment of DKA by a published protocol [36], [37]. Three patients received intravenous mannitol during the treatment of DKA based on the assessment by the attending of clinical signs suggesting early clinical cerebral edema, as previously described [36], [37]. All 17 patients had uneventful correction of DKA, frequently within 12 hours after the initiation of the treatment [37]. Laboratory results of the patients are depicted in Table S1. All patients had one or more positive islet cell autoantibodies (IAA, IA-2, and GAD65) (data not shown). Troponin I was not increased in any patient at either T1 (6–12 hrs) or at 24 hrs (data not shown).

**Table 1:** Demographic data for 17 patients in data set.

Patient No.	Age at First Data Set (years)	Disease Duration at First Data Set	Gender	Race	Diastolic Abnormality Group
1	13.25	1 d	M	C	No
2	14.42	5 y	F	AA	Yes
3	13.58	9 y	F	C	Yes
4	16.92	3 y	M	AA	Yes
5	11.42	3 y	M	AA	No
6	10.08	1 d	F	C	No
7	16.92	1 y	M	AA	No
8	16.33	1 d	F	AA	Yes
9	13.08	1 d	M	C	No
10	14.33	12 y	F	AA	Yes
11	11.58	1 d	F	AA	Yes
12	16.25	6 y	M	AA	Yes
13	15.25	7 y	F	C	No
14	9.67	1 d	F	AA	No
15	15.5	13 y	F	AA	Yes
16	10.75	5 y	M	AA	No
17	14.58	6 y	F	C	No
	**Average Age (SD) in years** 13.76 (2.37)	**Mean (SD) Duration in years** 4.12 (4.29)	**TOTAL** 7 Male: 10 Female	**TOTAL** 11 AA*: 6C	**TOTAL** 8 DA Group: 9 Non-DA Group

* Fisher's Exact chi-square analysis indicates a significant association between the Diastolic Abnormality and the African American race (p = 0.0319).

### 2. Cardiac Assessment

To assess cardiac parameters of patients with DKA, ECHO was performed at T1 (6–12 hrs) vs T2 (2–3 wks/ECHO baseline). The results are shown in Table 2. Heart rates (HRs) obtained during ECHO examinations were significantly elevated (p<0.0001) at T1 (106 bpm) vs T2 (78 bpm). Two dimensional LV fractional shortening was significantly higher at T1 compared with T2 but within normal range at both studies, indicating increased systolic performance with the increased adrenergic state. LV dimensions and volumes were significantly lower at T1 consistent with changes from dehydration and sinus tachycardia associated with DKA.

**Table 2:** Comparative analysis for ECHO variables that had significant differences for all patients at T1 (6–12 hours post admission) vs T2 (2–3 weeks/ECHO baseline), (N = 17 patients).

ECHO Variable	Echo T1 DKA Mean(SD)	Echo T2 Baseline Mean(SD)	Test Result * T (p)
RR Interval (msec)	566.47 (68.67)	771.47 (159.19)	−5.48 (<0.0001)
Mitral Valve E/A Ratio	1.49 (0.27)	2.15 (0.36)	−6.29 (<0.0001)
2D LV diameter Diastolic (cm)	3.98 (0.61)	4.44 (0.52)	−4.38 (0.0002)
2D LV diameter Systolic (cm)	2.38 (0.40)	2.81 (0.45)	−5.25 (<0.0001)
LV Area Diastolic (cm^2^)	13.12 (2.67)	16.23 (3.23)	−5.33 (<0.0001)
LV Area Systolic (cm^2^)	5.94 (1.60)	7.11 (2.05)	−2.88 (0.0054)
LV Volume Diastolic (cm^3^)	36.08 (10.54)	48.91 (14.91)	−4.70 (0.0001)
LV Volum Systolic (cm^3^)	10.93 (3.98)	14.28 (5.94)	−2.93 (0.0049)
2D SF%	39.88 (5.79)	36.91 (5.28)	1.80 (0.0452)
2D LV Wall thickness Diastolic (cm)	0.77 (0.12)	0.71 (0.13)	1.88 (0.0305)
2D Septum thickness (cm)	0.74 (0.12)	0.69 (0.12)	1.75 (0.0398)
LA diameter (cm)	2.31 (0.47)	2.80 (0.43)	−5.11 (<0.0001)
Aorta diameter (cm)	2.42 (0.34)	2.18 (0.33)	3.14 (0.0031)

* Statistically significant results p<0.05.

### 3. Diastolic Abnormality

**Figure 1:**
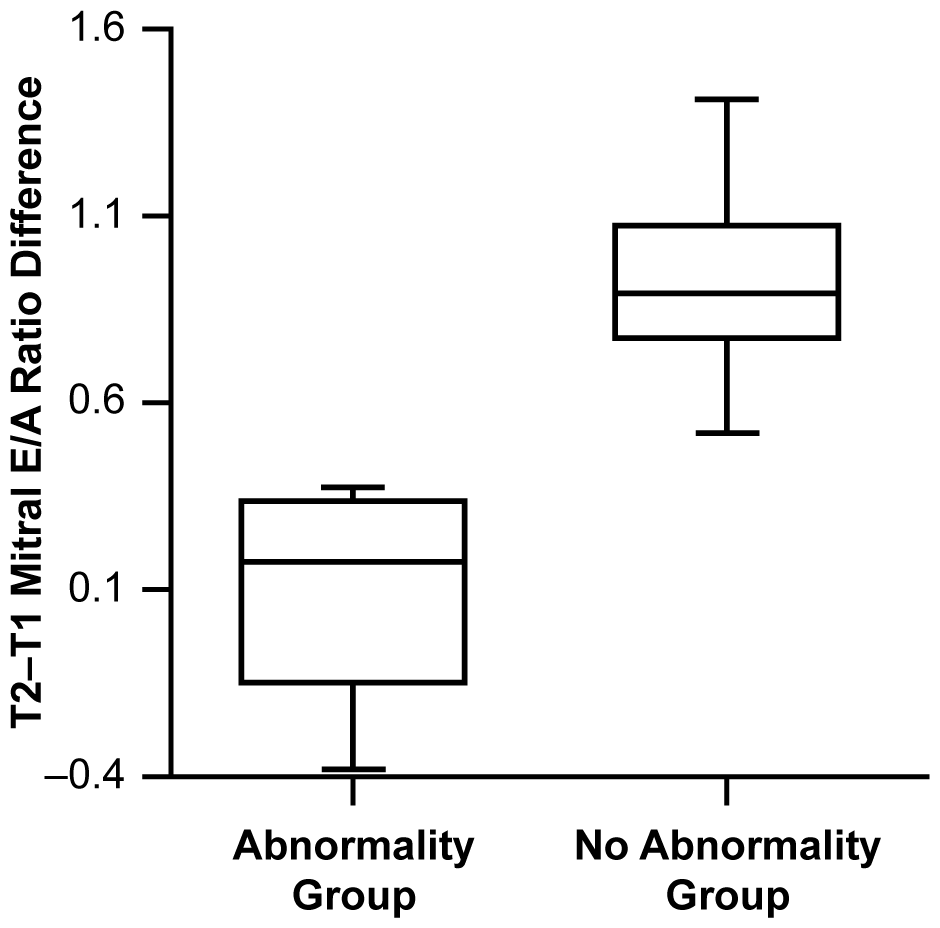
Figure 1 shows that the difference in mitral E/A at T2 (Baseline) minus E/A at T1 (DKA) was significantly less (Z = −3.1115, p = 0.0019) for the group of 5 patients with DA at T1 compared to the other 12 patients. HRs were not different in these 5 patients compared to the other 12 patients at T1 or T2.

Diastolic function by mitral valve Doppler E/A filling ratio showed a significantly lower mean mitral E/A filling ratio at T1 compared with T2 for all 17 patients (Table 2). This is a normal finding since mitral diastolic E/A ratio shortens progressively with faster HRs [40], [41] and all patients had sinus tachycardia at T1. A comparison of the difference in the E/A ratio between T2 and T1 indicates that in five patients there was not a normal shortening (decrease) of the E/A ratio during T1 compared with the baseline at T2. This lack of normal diastolic adaptation to sinus tachycardia identified this subgroup to have an acute diastolic abnormality (DA) during DKA (Figure 1).

**Figure 2:**
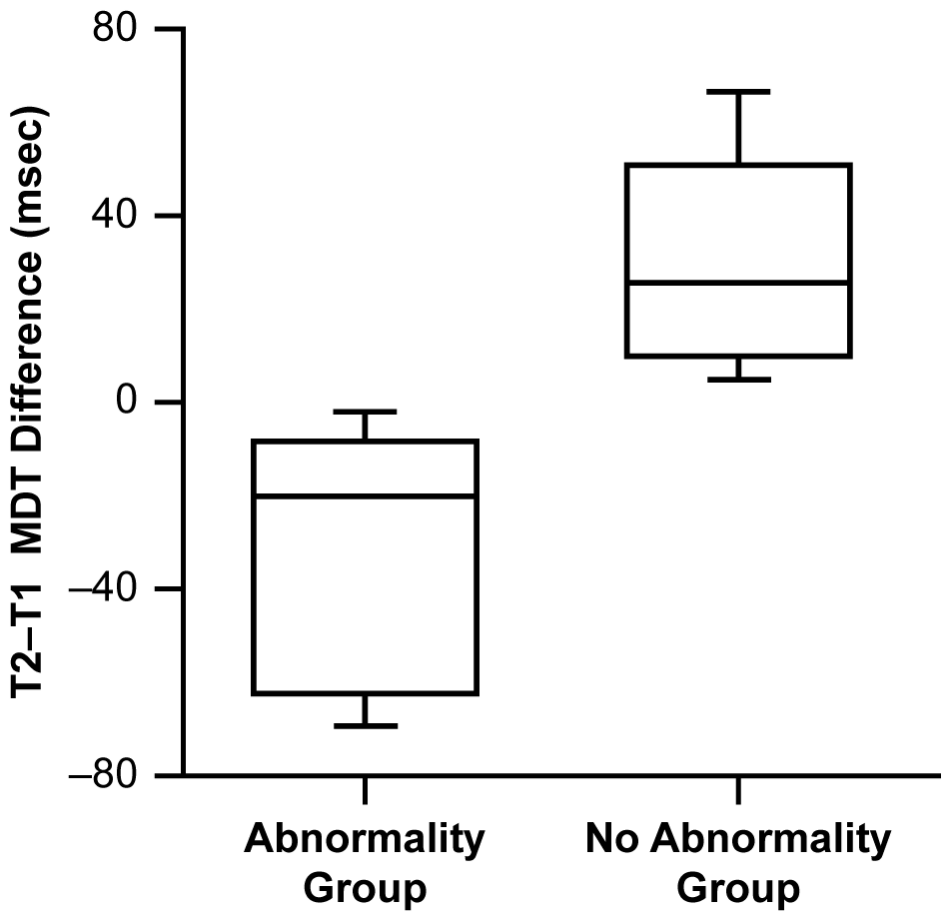
Figure 2 shows that the difference in MDT at T2 minus T1 for the patients identified with mitral DA. The magnitude of the difference was statistically smaller (Z = −3.2684; p = 0.0011 : T1 not shorter than T2) in the DA group patients implying abnormal adaptation to sinus tachycardia at T1.

Mean mitral deceleration time (MDT) was shorter at T1 (mean = 154 msec) compared with T2 (mean = 166 msec) in the group as a whole. This is also consistent with a normal response to the shortening in diastolic filling time during sinus tachycardia. However, in six patients the MDT was not shorter at T1 compared with T2, despite the significantly higher HR at T1. This represents a significant association for abnormal MDT adaptation to sinus tachycardia at T1 for these 6 patients (p = 0.0345). Analysis of the difference between MDT at T1 and T2 indicated that the magnitude of the difference was statistically smaller (MDT did not shorten at T1 compared with T2) in these six patients (p = 0. 0011). This relationship is depicted in Figure 2. Three patients showed this abnormal response to an increased HR in both of these diastolic parameters: mitral E/A ratio and mitral DT. Therefore eight (8) patients had one or both of these abnormal diastolic changes with sinus tachycardia and were identified as having diastolic dysfunction or diastolic abnormality (DA) compared with the other 9 patients with no demonstrable acute DA (non-DA) at T1. No significant difference in HR values were present at T1 between the 8 DA patients versus the 9 non-DA patients supporting that a true diastolic HR adaptation abnormality was present during DKA in the DA patients.

### 4. Diastolic Abnormality vs No Abnormality

The 8 DA patients versus the 9 non-DA patients showed slightly greater mean HR values (DA at 107 bpm; non-DA at 104 bpm), but no significant difference at T1. Mean echocardiographic changes for the patients after grouping by the presence of DA or non-DA are summarized in Table S2. At T1, the DA patient group had significantly thicker left ventricular wall and septum, larger left atrial volumes and longer mitral deceleration time corrected for HR (MDT HRc) compared to the non-DA group. In addition, the mean mitral E/A ratio for the DA group was lower at T2 compared to the non-DA group, with no significant differences at T1.

Although gender, age and duration of diabetes had no statistically significant relationship with DA, there was a significant association between the AA race and DA group (p = 0.0319). Also, a significant percentage of AA patients presented with DA having a shorter duration of T1DM (p = 0.0228). There was no significant race or DA group differentiation with HbA1c at T1 or T3.

### 5. Inflammatory Cytokines

To examine levels of chemokines/cytokines, blood samples were collected at: T1 at 6–12 hrs; T2 at 2–3 wks; and T3 at 3 mon (cytokine/chemokine baseline). Twenty seven of the thirty nine cytokines had the highest value at T1 (p = .0014). Ten of the cytokines/chemokines (26%) had a statistically significant variation in mean values across time (Table 3). In addition we observed statistically significant correlations between admission chemistries and cytokines at T1 and T3 (Table S3). ANOVA comparisons of cytokines/chemokines levels sorted by duration of T1DM indicated no significant differences in concentrations between newly diagnosed and those with longer duration (data not shown).

**Table 3:** Repeated Measures ANOVA results (F-ratio and probability) for cytokines with significant differences across time. T1 (6–12 hours post admission); T2 (2–3 weeks); T3 (3 months).

Cytokine	F-Ratio	Probability	Sample Time:	Mean (SE)
EOTAXIN	7.73	0.0018	T1	52.47 (4.94) ^++^
			T2	43.17 (4.19)
			T3	38.54 (4.52)
GM-CSF	6.00	0.0064	T1	77.68 (14.26)* ^++^
			T2	48.32 (10.60)
			T3	43.93 (11.14)
IFN-gamma	5.85	0.0071	T1	42.43 (12.81)* ^++^
			T2	30.22 (10.15)
			T3	24.99 (8.50)
IL-6	5.45	0.0092	T1	26.29 (11.14) ^++^
			T2	20.43 (8.79)
			T3	17.57 (8.91)
IL-8	13.21	<0.0001	T1	16.12 (4.16)* ^++^
			T2	9.38 (3.46)
			T3	6.40 (2.73)
IL-17	5.45	0.0096	T1	17.05 (6.09) ^++^
			T2	10.81 (4.95)
			T3	9.71 (4.10)
CCL7 (MCP-3)	5.16	0.0119	T1	19.18 (2.78) ^++^
			T2	15.65 (2.47)
			T3	12.80 (2.17)
MDC	11.92	0.0001	T1	1034.45(81.19)* ^++^
			T2	1456.85 (106.93)
			T3	1303.77 (123.44)
sIL-2ra	4.08	0.0271	T1	25.19 (11.12)^++^
			T2	2.97 (2.41)
			T3	1.38 (1.22)
VEGF	3.38	0.0473	T1	262.57 (62.51)^++^
			T2	209.20 (66.48)
			T3	181.62 (43.48)

* - Mean (T1) significantly different (p<0.05) from (T2) mean; ++ - Mean (T1) different (p<0.05) from mean cytokine baseline measurement (T3). N = 17 subjects.

### 6. Diastolic Abnormality and Cytokine Interactions

**Figure 3:**
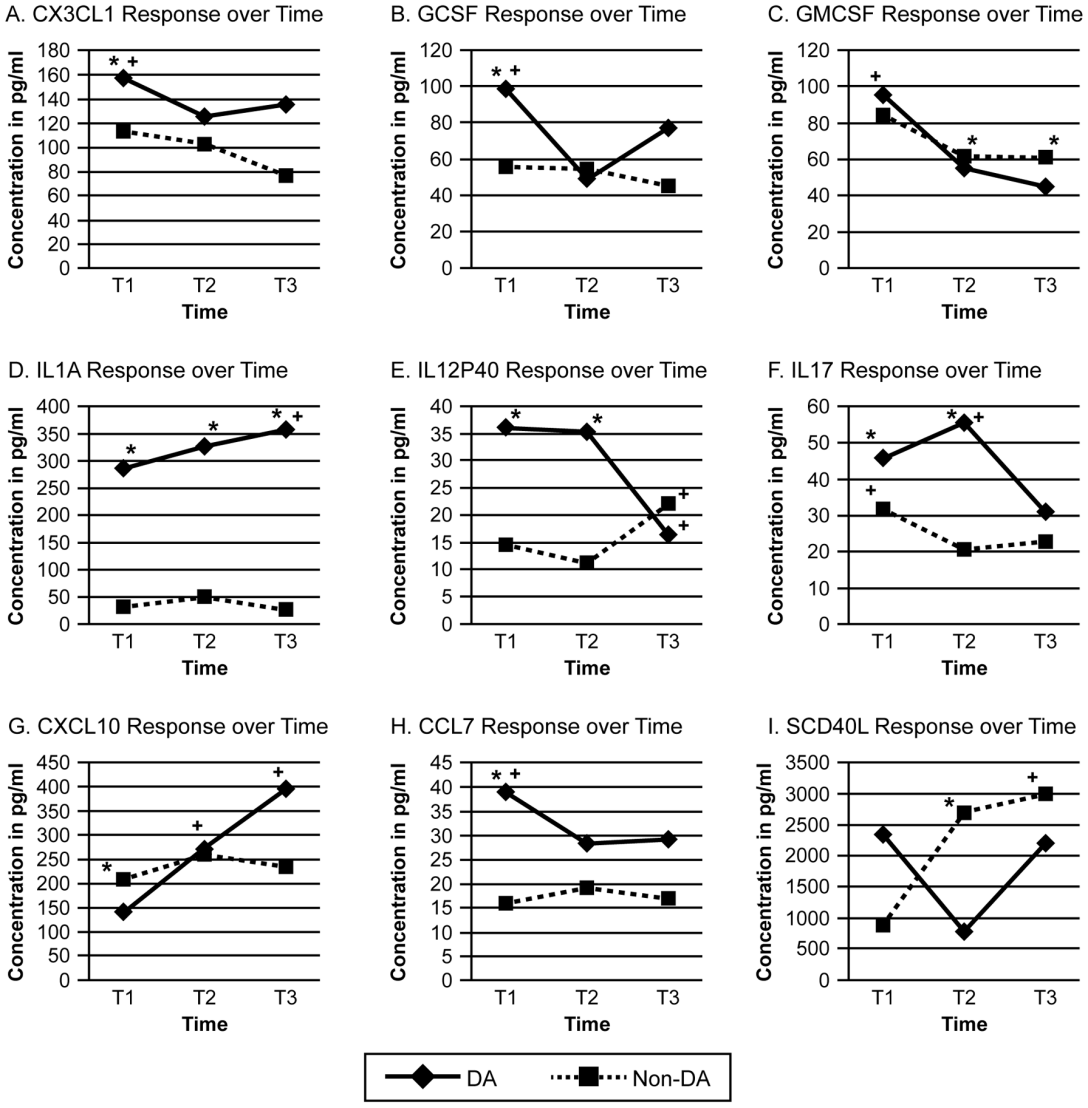
a–i. *Identifies statistically significant (p<0.05) differences between DA and Non-DA groups for the same time point (5a–i). + identifies statistically significant (p<0.05) differences within DA or Non-DA groups across time points (5a–i). T1 (6–12 hrs post admission); T2 (2–3 wks); T3 (3 months).

A two factor ANOVA with repeated measures on time indicated that nine of the 27 measured cytokine/chemokines displayed mean values that were significantly different across time depending on whether the patients displayed an ECHO diastolic abnormality (DA group) or did not (non-DA group) at T1 (Figures 3a–i). The mean GCSF, IL-1α, CCL7 (MCP3), CX3CL1 (Fractalkine), IL-17, GM-CSF, IL-12p40 and sCD40L concentrations were increased at T1 in the DA group compared to the non-DA group. Conversely, the mean CXCL10 (Interferon gamma-induced protein 10, IP-10) concentration was decreased in the DA group at T1. At T3 there was no difference in 6 of the cytokine/chemokines GCSF, CCL7(MCP3), CX3CL1 (Fractalkine), IL-17, CXCL10 (IP10), sCD40L between the DA and non-DA group. IL-1α remained higher in the DA than in the non-DA group. GM-CSF and IL12p40 values were lower in the DA than in the non-DA group. Additionally, at T1 (6–12 hrs), we observed statistically significant correlations between the DA group determinant ECHO variables of MDT or mitral E/A ratio and the cytokines IL-12p40, sCD40L, CXCL10 (IP10), IL17, and GMCSF (Table 4).

**Table 4:** Significant Spearman correlations between cytokines displaying association effects and DA determinant ECHO variable Mitral E/A and Mitral Deceleration Time (MDT).

Relationship @ T1	Spearman Correlation Coefficient	p value*
MDT: IL12P40	r. = −0.7233	p = 0.0078
MDT: sCD40L	r. = 0.5360	p = 0.0323
Mitral E/A: IP10 (CXCL10)	r. = −0.6158	p = 0.0111
Mitral E/A: IL12P40	r. = 0.5440	p = 0.0293
Mitral E/A: IL17	r. = 0.5068	p = 0.0451
Mitral E/A: GMCSF	r. = 0.6135	p = 0.0196

Relationships for the DA group determinant ECHO variables and cytokines were calculated.

* Only statistically significant (p<0.05) correlations are tabulated. All comparisons are at T1. An inverse relationship (−r value) implies higher cyotikine concentrations at shorter/normal mitral deceleration time (MDT) and mitral E/A times. Direct relationship (+r value) implies higher cytokine concentrations at longer/abnormal MDT and mitral E/A ratios. The strongest correlation is IL12p40 and MDT (r. = −0.7233), accounting for 52% of the variation noted between the two variables.

### 7. Hypertension

**Figure 4:**
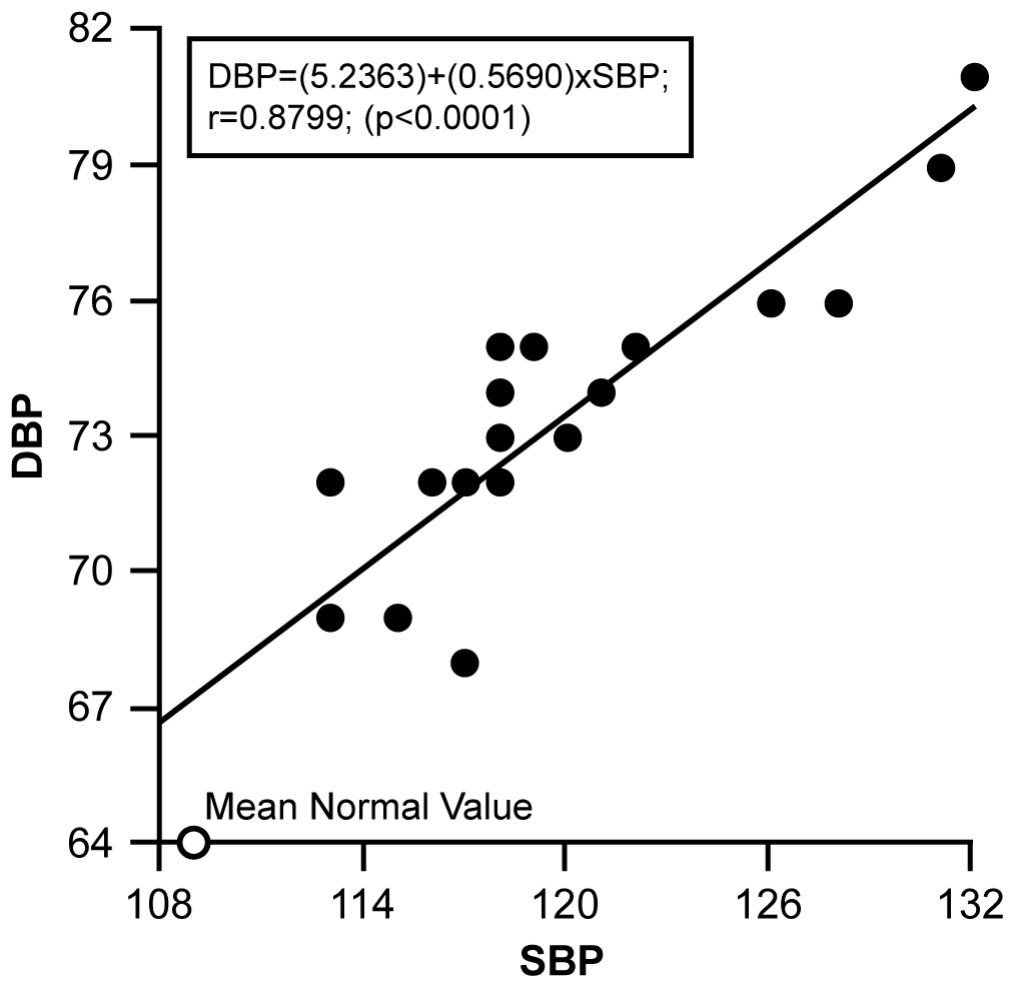
Figure 4 shows the linear regression plot for SBP and DBP over time. Mean age related normal value is plotted in relation to patient SBP/DBP values for comparison.

Blood pressures were recorded for the 17 patients at 19 time points that included 16 measurements over the first 24 hrs of treatment; at discharge (48 hrs); at 2–3 wks (baseline ECHO) (T2); and at 3 mon (baseline cytokine/chemokine) (T3). An elevated BP was present on admission for 15 of the 17 patients (p = 0.0023). A direct correlation between mean SBP and DBP was established with a correlation coefficient r = 0.8799, (p<0.0001). Figure 4 depicts the linear regression line relating mean SBP and DBP using the 19 points with the mean normal SBP and DBP value plotted as it relates to the elevated patient mean SBP and DBP values. The regression equation is DBP = (5.2363)+(0.5690)×SBP. There were no statistically significant correlations between duration of T1DM and either SBP or DBP.

**Figure 5:**
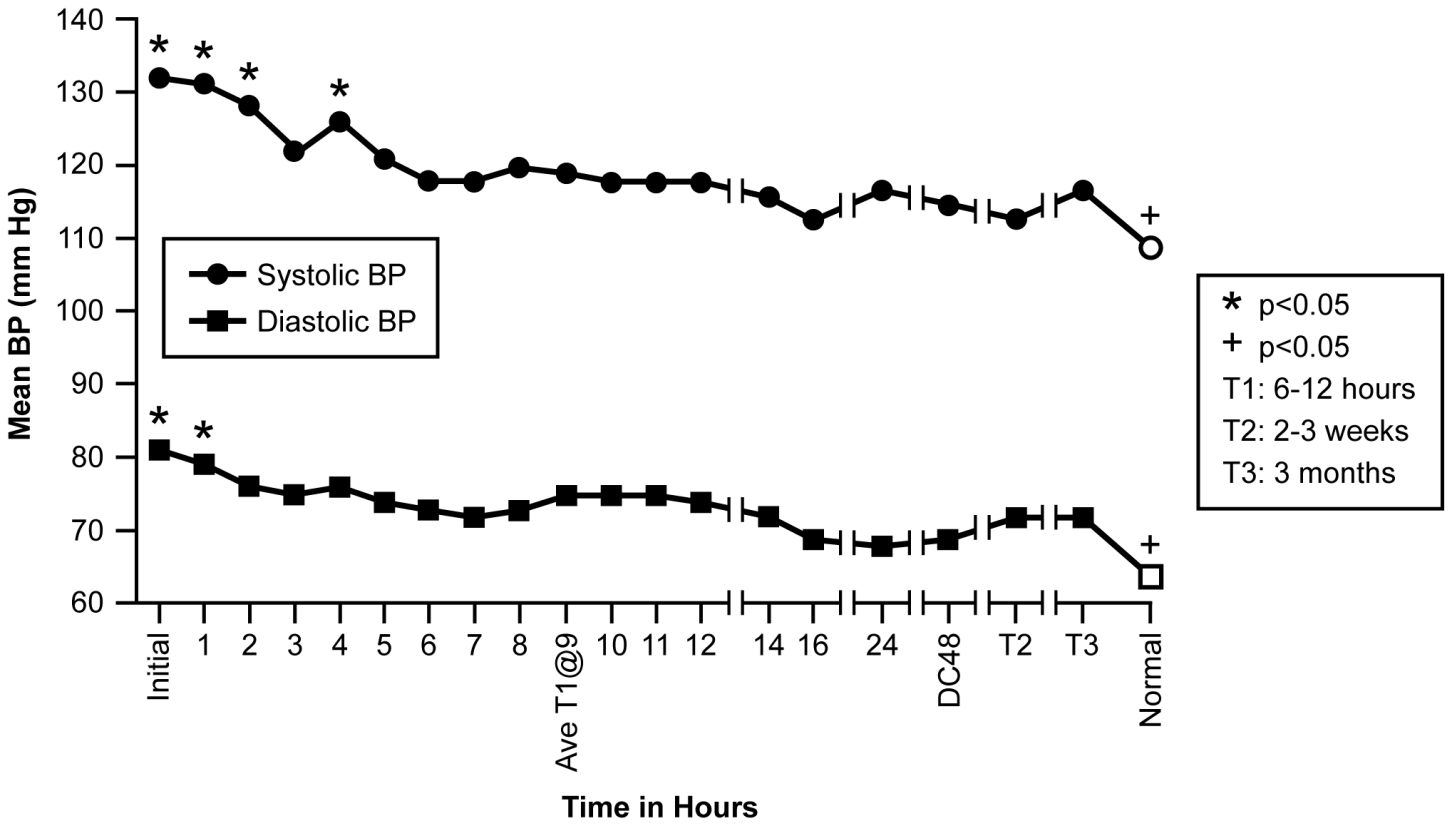
Figure 5 shows the mean SBP (mmHg) and DBP (mmHg) across 19 time points. For comparison, age specific normal measurements are included as the last measurement on the X-axis.

Statistically significant differences in mean SBP, DBP across the BP time frame were established with repeated measures ANOVA (SBP Mean = 120 mm Hg; F = 4.73, p<0.0001; DBP Mean = 73 mm Hg; F = 3.29, p<0.0001). Comparison of the mean SBP and DBP measures indicate significant differences (p<0.05) for early sample times compared to later times, as well as the mean age related normal BP measurements (Figure 5). The initial, T2 and T3 SBP and DBP were all significantly greater than normal reference BPs [42] (indicating these patients were hypertensive during the study). Intravenous mannitol for three patients had no statistical effect on the group BP measurements (p = 0.7322).

### 8. Diastolic Abnormality and BP Relationships

**Figure 6:**
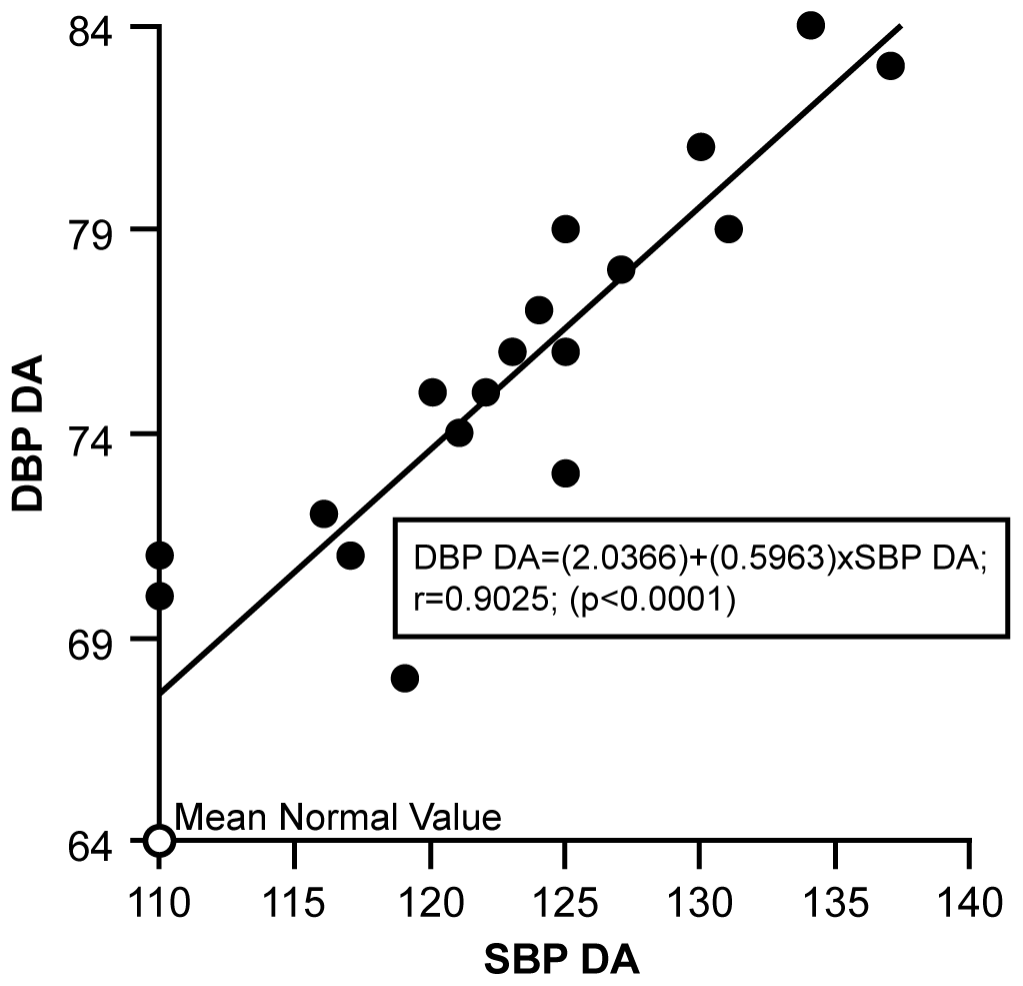
Figure 6 shows the linear regression plot for SBP-DA and DBP-DA over time. Mean age related normal valued is plotted in relation to patient SBP-DA/DBP-DA values for comparison.

**Figure 7:**
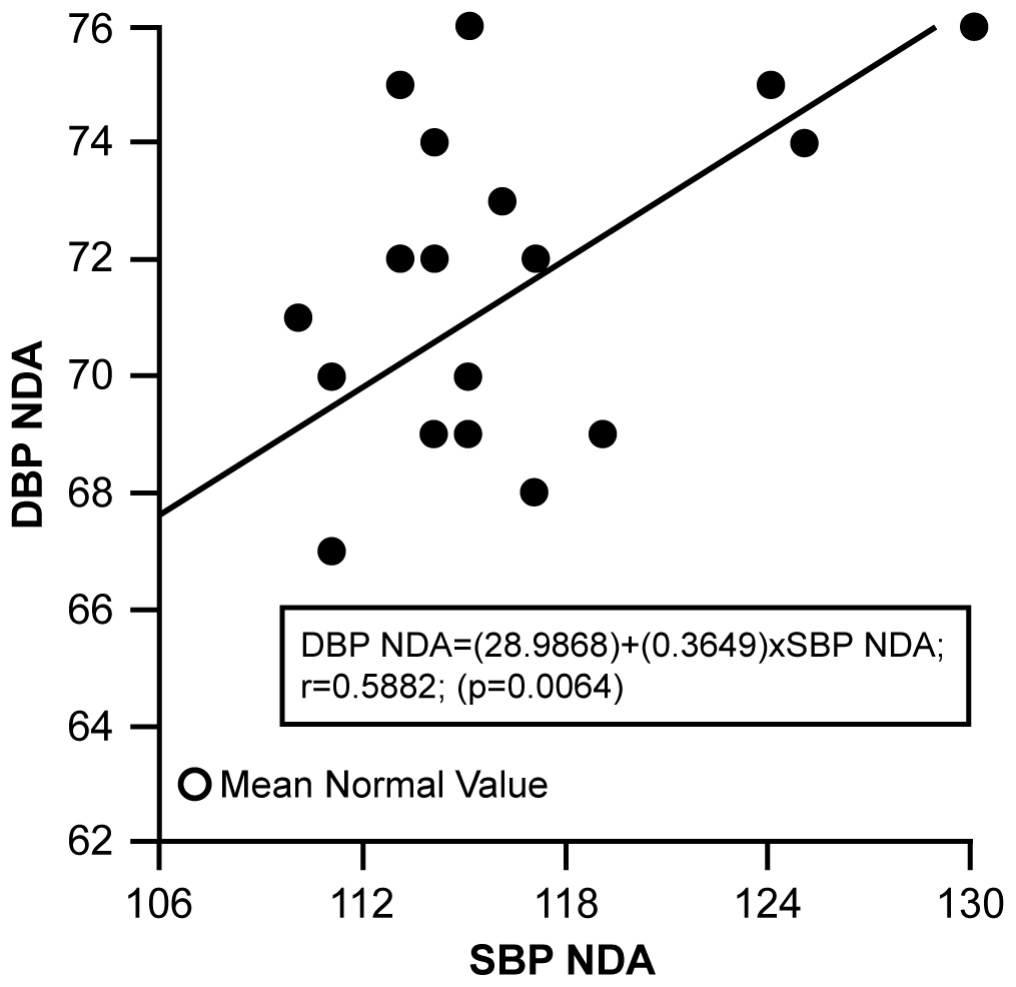
Figure 7 shows the linear regression plot for SBP-Non DA and DBP-Non DA over time. Mean age related normal valued is plotted in relation to patient SBP-Non DA/DBP-Non DA values for comparison.

Grouping the 19 BP time points according to ECHO evidence of DA resulted in the following observations based on linear regression/correlation analyses using the variables SBP-DA or DBP-DA and SBP-non-DA or DBP-non-DA. Figure 6 relates the mean SBP-DA to the DBP-DA, with the mean normal SBP-DA and DBP-DA value plotted as it related to the elevated patient mean values. The direct correlation between mean SBP-DA and DBP-DA is 0.9025, (p<0.0001). The value of R^2^, the proportion of the variation in DBP-DA that is accounted for by variation in SBP-DA, is 0.8146 or 82%. The regression equation is DBP-DA = (2.0366)+(0.5963)×SBP-DA. Figure 7 relates the mean SBP-non-DA to the DBP-non-DA, with the mean normal SBP-non-DA and DBP-non -DA value plotted as it related to the elevated patient mean values. The correlation between mean DBP-non-DA and SBP-non-DA is 0.5882, (p = 0.0064). The value of R^2^, the proportion of the variation in DBP-non-DA that can be accounted for by variation in SBP-non-DA, is 0.3460 or 35%. DBP-non-DA = (28.9868)+(0.3649)×SBP-non-DA. Figures 6 and 7 indicate that the two regression lines are different, in variability, slope and intercept. The non-DA regression in Figure 7 is more variable (S.E. Reg. = 3.488) than the DA regression (S.E. Reg. = 2.425) in Figure 6. A general linear test (GLT) comparing the intercepts and slopes of the non-DA regression line to the DA regression line was significant (p = 0.033), indicating a significant difference between the DA group and the non-DA group SBP/DBP relationships. The DBP of the DA group is strongly related to the SBP (R^2^ = 0.8146), elevated SBP is related to elevated DBP. However, the SBP and DBP of the non-DA group are less related to each other (R^2^ = 0.3460), with strong relationships between SBP and DBP at the highest measures of SBP.

**Figure 8:**
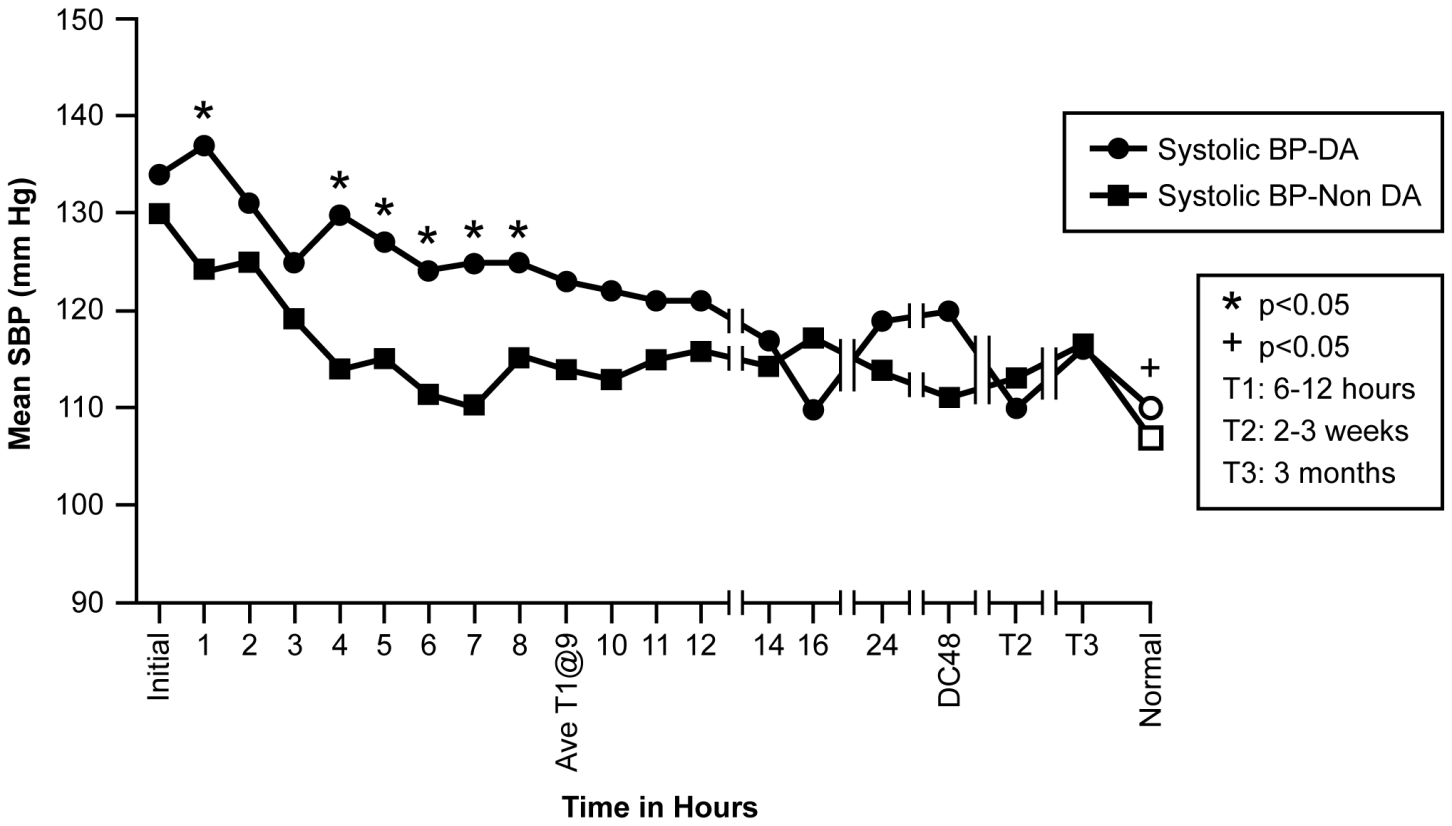
Figure 8 shows mean SBP (mmHg) for groups DA and Non-DA across 19 time points. Age specific normal measurements are included as the last measurement in on the X-axis.

**Figure 9:**
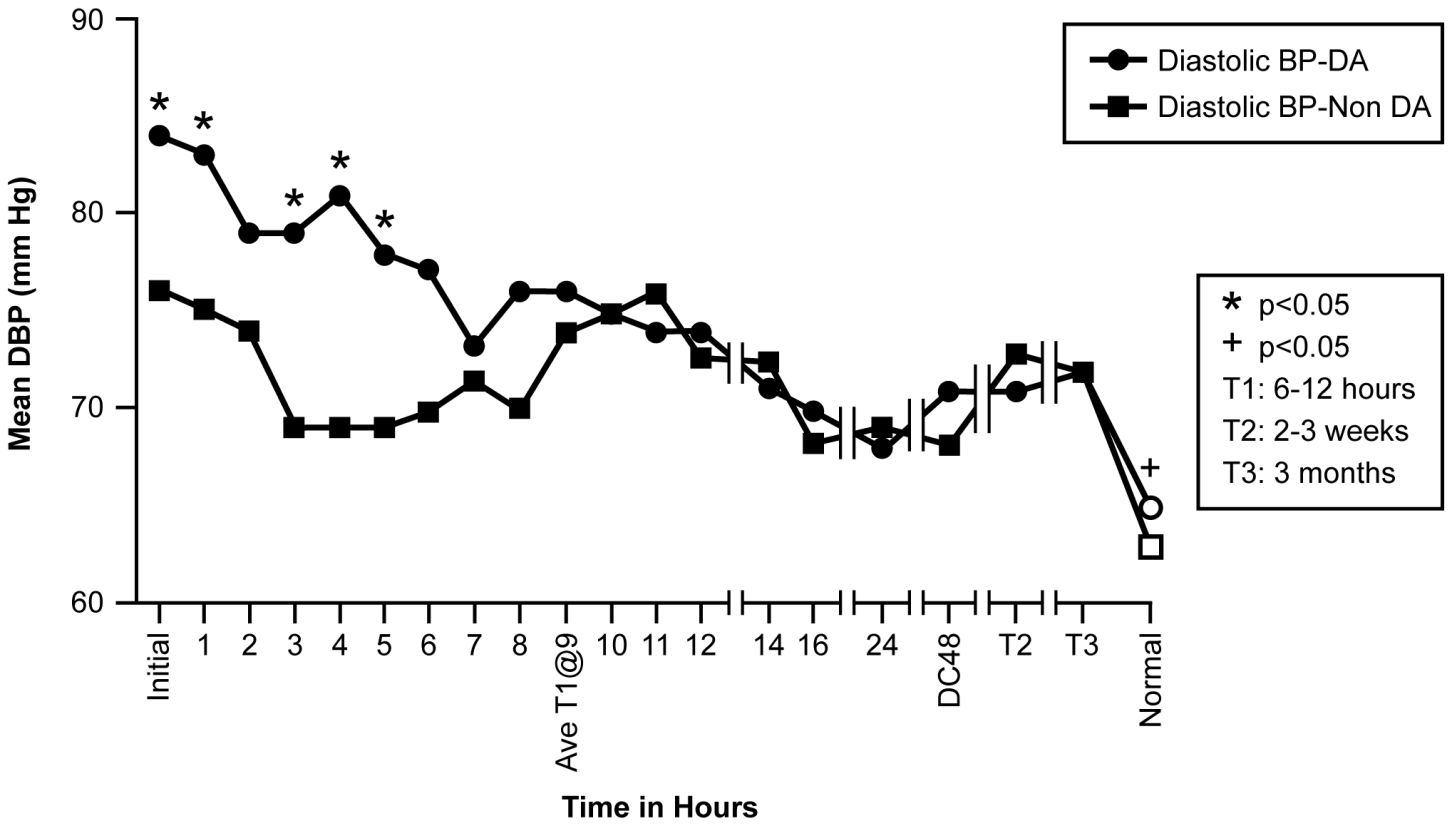
Figure 9 shows mean DBP (mmHg) for groups DA or Non-DA across 19 time points. Age specific normal measurements are included as the last measurement on the X-axis.

Examination of the 19 BP time periods grouped according to ECHO evidence of DA resulted in the following observations based on repeated measures ANOVA analysis, and depicted in Figures 8 & 9. Although the SBP trend identified for all 17 patients collectively did not change from early measures of SBP being significantly greater than later measures of SBP, differences became evident after grouping the subjects by DA or non-DA group. During hrs 4, 5, 6, 7, and 8, the mean DA group SBP was significantly greater (p<0.05) than the non-DA group SBP (Figure 8). The DBP trend established for all 17 patients also did not change from higher values early to lower values later. However with grouping, the significant differences between the DA group and the non-DA group were evidenced earlier specifically during the initial measurement through hour 5 (DA range: 84–78 mmHg; non-DA range: 76–68 mmHg), followed by a close agreement for the remaining time measures (Figure 9).

In summary, ECHO and cytokine/chemokine comparisons indicated significant differences over time in patients with DKA. Further, the ECHO analysis identified a group of patients displaying a DA. Grouping patients according to the presence or absence of a DA and testing for interaction effects with cytokines/chemokines indicated that 9 of the cytokines/chemokines responded differently over time. Blood pressure analysis also indicated significant differences over time as well as a differential response depending on the presence of the DA. There is a significant association between both AA race and duration of T1DM with DA.

## Discussion

Previous ECHO studies in young T1DM patients focused on assessing diastolic function during a stable metabolic state at one time and were comparing them with age-matched controls [6], [7], [43], [44]. The most common abnormality of diastolic function found in the prior studies of children with T1DM was a lower mitral E/A ratio; that is the comparison of the ECHO Doppler rapid LV filling (E) to the velocity of the late atrial component of LV filling (A). In contrast, our ECHO studies were performed during the acute metabolic crisis and immunologic dysregulation of DKA, and were then compared with the ECHO following correction of DKA in the same patient. The mitral E/A ratios have predictably been shown to decrease as HR increases [41]. Mitral deceleration time (MDT), another common measure of diastolic LV function, is a measure of the decay slope of the LV rate, and also decreases with the rapid filling velocity of the increased HR. Eight of our patients lacked these normal responses to sinus tachycardia in one or both of these parameters when compared with their (baseline) ECHO at 2–4 weeks after DKA (T2). Since the sinus tachycardia during DKA was no different in these 8 patients than in the other 9 patients, the 8 patients were considered to have an abnormal diastolic (DA) response to sinus tachycardia and were studied in relation to the systemic inflammatory cytokines/chemokines (6–12 hrs, during DKA treatment) T1, (2–4 wks post discharge) T2, and (at 3 mons after DKA) T3. Consistent with these diastolic changes, the DA group had larger LA volumes than the non-DA group at T1 consistent with impaired diastolic relaxation/filling. The Doppler flow abnormalities we identified during DKA (T1) indicated an abnormal diastolic function in relation to the baseline ECHO study (T2).

Although our initial goal was to study diastolic function during DKA, we also found lower baseline mitral E/A ratios in the DA patients at T2, compared to the non-DA patients. This is consistent with prior studies of diastolic function in children and adolescents with stable T1DM [7], [44]. Importantly, Wojcik et al., reported correlations between ECHO abnormalities and HbA1c values from the two years prior to the ECHO [44]. As in our study, no correlation was found with the HbA1c at the time of the ECHO. The correlations of diastolic dysfunction with: 1) HbA1c values considerably prior to the ECHO; 2) the longer durations of diabetes associated with diastolic dysfunction [7]; and 3) the improvement of the DA following correction of DKA suggest a diastolic adaptation prior to the development of clinical dilated cardiomyopathy, possibly as the result of a subclinical chronic inflammatory pattern in a subset of genetically predisposed patients.

Markers of oxidative stress and the duration of T1DM in rodent models support early abnormal diastolic function prior to contractile abnormalities [45], [46]. However, previous ECHO studies in children with T1DM and stable metabolic control differ as to the effect of metabolic control on diastolic function [6]–[8], [43], [44]. Although both hyperglycemia and ketoacidosis were considered sources of oxidative and inflammatory stress [16], [47], we found no correlation between the admission BG, HbA1c and pCO2, nor between the (T1) BG and pCO2 and the DA. This supports the view that the metabolic risk factors for DCM require a longer interval to impact diastolic function, as suggested by Wojcik's et al., study [44]. This finding does not rule out an acute additive effect of SIR.

The present study extends observations of the activation of the immune system resulting in a SIR that involves upregulation of inflammatory cytokines during the treatment of DKA [26]–[28], [30]. Nine of the cytokine/chemokines that were increased at the time of the DA 6–12 hours into treatment (T1) are involved in the pathogenesis of EAM [48] and cardiomyopathy [49], [50]. Four of these nine interactive cytokines (GM-CSF, G-CSF, IL12p40, IL17) are associated with Th17 cell response. Autoimmune disorders, including T1DM, were thought to be primarily driven by a Th1 response. This was challenged by the discovery of Th17 cells and their implication in the pathogenesis of multiple autoimmune diseases [51]. IL-6 and IL-1β induce retinoic acid receptor-related orphan receptor C2 (RORC2) and IL-17 activation in memory T cells, whereas TGF-β and IL-21 in combination induce differentiation of Th17 cells from naive T cells [52]. Both IL-6 and IL-1β were increased in our previous DKA study when samples were collected prior to treatment [26]. Children with T1DM had increased IL17 transcript in their memory T cells. In addition, RORC2 and IL22 were produced by activated as well as memory T cells from T1DM children [53]. There are conflicting results about role of IL17 in the pathogenesis of diabetes in the NOD mouse model. IL-17A deficient NOD mice do not develop diabetes, while blocking IL-17 with monoclonal antibodies protected young NOD mice from diabetes development [54], [55]. The potential explanation of this discrepancy is an additive effect of IL17F to diabetes pathogenesis, since the blocking antibodies were not specific to IL17A. Diabetes can be also transferred by Th17 cells injected into NOD SCID recipients [56]. New onset T1DM patients have an increased proportion of IL-17A secreting T cells in their blood [57], although we did not find any correlation of increased Th17 cytokines during DA with the new onset of T1DM compared to previously diagnosed T1DM patients. IL17A is essential for progression to dilated cardiomyopathy [58] and is also involved in the pathogenesis of acute and chronic vasculitis and angiotensin II-induced hypertension [59]–[61].

GM-CSF, key hematopoietic factor, has recently been connected with the IL23-IL17A inflammatory pathway. Recent evidence suggests that many of the inflammatory functions of Th17 cells are actually attributable to GM-CSF [62]. Levels of G-CSF are increased in myocarditis/cardiomyopathy and acute myocardial infarction [63]; G-CSF has been described as another major component of Th17 host defenses. The control of granulocytic responses comprises a major effector arm of Th17 immunity by expanding the neutrophil compartment and chemotactic recruitment of neutrophils by CXCL1/KC and CXCL8/IL8 [64]. IL-12p40 functions as a bioactive and regulatory component of IL-12, a chemoattractant for macrophages, and provides negative feedback by competitively binding to the IL-12 receptor [65]. Of greatest importance in DKA, the IL-12p40 subunit combines with the p19 protein to form IL-23 [66], driver of Th17 cell expansion and maintenance [67], [68]. IL-23 is involved in the pathogenesis of autoimmune myocarditis [69] via the expression of Th17 cells [70]. Two of the nine interactive cytokines/chemokines (Fractalkine and MCP3) that we found to be associated with diastolic dysfunction in DCA are related to monocyte trafficking, an important step in the pathogenesis of experimental autoimmune myocarditis (EAM) [71]. Fractalkine is a highly expressed adipochemokine of monocytes and non-cardiomyocytic non-inflammatory cells in human inflammatory cardiomyopathy [72], [73]. MCP3, one of three members of a subfamily of beta-chemokines, activates a range of cell types [74] and is an attractant for human CD4^+^ and CD8^+^ T lymphocytes [75]. Reduced degradation of MCP-3 increases myocardial inflammation in experimental myocarditis [76], [77]. Each of the other three interactive cytokines (IL-1α, sCD40L, and IP-10) is also involved in experimental and/or clinical cardiovascular disease [78]–[82]. Further studies are warranted to determine if these nine cytokine/chemokines are candidate biomarkers for DCM. The importance of such markers in diabetic cardiovascular disease has been reported by Schram et al. [83].

Suys et al. reported significant ECHO changes in female children in comparison to males with T1DM [6]; however, we found no association between gender, age or duration of T1DM with DA. We did identify a significant association between race (AA) and DA (p = 0.0319), suggesting a genetic predilection and possible differences in cardiac response. The study by Ness et al. (2004) [84] is important relative to the association between AA children and the cytokines associated with DA. They reported the differential distribution of allelic variants in cytokine genes among non-diabetic AA and Caucasians, specifically that AA females are significantly more likely to carry allelic variants that up-regulate pro-inflammatory cytokines, and also to have genotypes known to down-regulate the anti-inflammatory IL-10.The lack of a pre-treatment sample prevented the correlation of the anti-inflammatory cytokine IL-10, which protects against EAM [85], with DA. Both our previous DKA study [26] and that of Karavanaki et al. [27], reported pre-treatment elevations of IL-10 and decreasing concentrations with the initiation of treatment.

Based on no difference between the HR of DA and non-DA groups at T1, and considering HR as a marker of hydration, dehydration does not appear to be a factor in the DA. A statistical difference did exist between the degree of hypertension of the DA group and the non-DA group through the first 8 hours of treatment, after which a difference in the BPs of the two groups remained but was not statistically significant. It is important to note that the mean BPs for DA and non-DA at T2 and T3 were statistically elevated in relation to age-matched normal control values [86]. Our prospective findings are in keeping with the retrospective study by Deeter et al. [87] of a slight but sustained BP elevation following correction of DKA. Linear regression plots between the SBPs and DBPs and also between SBPs-DA and DBPs-DA have strong correlations, and thus agree with Deeter et al. [87] of no increases in DBP without increases in SBP. Even though there was no significant difference in the BPs between DA and non-DA patients at T1, the uncertain duration of BP elevation prior to admission precludes determining an impact of BP on mitral E/A and/or MDT. In a canine study of methoxamine-induced acute systolic BP elevation of 30 mm or greater, there was no acute effect on E/A ratio, when heart rate was kept constant by pacing, and a decrease in MDT suggesting that our observed changes in these parameters in the DA patients were not due to BP elevation [88].

Our study was not intended to evaluate the relationship between acute changes in cytokines/chemokines and BP. However, in addition to the increased secretion of counter-regulatory hormones in DKA [89] several other perturbations are candidates for mediating pre-treatment hypertension: 1) insulin resistance and endothelial dysfunction; 2) the increased oxidative stress caused by hyperglycemia [90], [91]; 3) and the ability of acetoacetate to increase the expression of the vasoactive peptide ET1 from capillary endothelial cells [92]. The literature also supports a role for inflammatory cytokine stimulation of the hypothalamic-pituitary-adrenal axis [93], [94] and adrenal medullary chromaffin cells [95]. In contrast to the recognized anti-inflammatory effect of insulin on endothelium in critical illnesses [96]–[98], our results confirm observations of increased inflammatory cytokines/chemokines during intravenous insulin [26], [27], [30]. Regarding the relationship of the SIR and the possible pathogenesis of myocarditis [99], it is important to note that two metabolites of poorly controlled diabetes-hyperglycemia [100] and free fatty acids (FFA) [101] amplify toll-like receptors (TLR) in monocytes and results in the potential for: TLR-4-mediated myocardial apoptosis and DCM [102]; and the enhancement of the expression of anaphylatoxin C5a [103] and other complement peptides that are increased systemically during DKA and its treatment [28]. C5a, in turn, could increase IL-17 and other inflammatory cytokines [104]. The involvement of IL-17 in the pathogenesis of EAM and dilated cardiomyopathy [58] and its potential involvement in DCM would be analogous to the insult of acute burn that activates leukocyte TLRs, and the resulting production of numerous cytokines/chemokines, including effectors in burn cardiomyopathy [105]–[108].

Finally, transient cardiogenic compromise has been suggested as an explanation for the subclinical interstitial pulmonary edema (IPE) that occurs prior to treatment of DKA, is accentuated during treatment [109]–[111] and corresponds to the time interval of SIR [26], [27]. The diastolic abnormality with increased LA volume and increased pulmonary venous pressure could contribute to subclinical IPE. The logistical limitation of obtaining a pretreatment ECHO and a cytokine/chemokine sample precludes determining the onset of DA and thus certainty of a relationship between DA and IPE. Based on the SIR during the same time interval in the treatment of DKA [26]–[28], [30] as the increase in IPE [111], inflammatory cytokines/chemokines could be independent candidates for pulmonary epithelial perturbation and IPE.

## Conclusions

This study is the first to report an acute DA associated with the systemic inflammatory cytokines/chemokine response in a subset of young T1DM patients during the acute metabolic and immunologic stress of DKA. This does not rule out the role for a metabolic insult in the pathogenesis of DCM. Further studies are required to determine whether the acute SIR of severe DKA produces a subclinical autoimmune myocardial insult as the result of individual cytokines, or a particular cytokine pattern, which could progresses asymptomatically to DCM [112].

## Supporting Information

Table S1 **Repeated measures T-tests for blood cell types and chemistries.**
(DOCX)

Table S2 **Echocardiography variable comparative analysis for DA (N = 8) and Non-DA (N = 9) groups at T1 (6–12 hrs post admission) or T2 (2–3 weeks/ECHO baseline).**
(DOCX)

Table S3 **Statistically significant Spearman correlations between admission chemistries including BG, HbA1c and cytokines at T1 (6–12 hours) and at T3 (3 months post admission).**
(DOCX)
