# Supplementary material for: Increased Systemic Th17 Cytokines Are Associated with Diastolic Dysfunction in Children and Adolescents with Diabetic Ketoacidosis
Source: PLoS One. 2013 Aug 27;8(8):e71905. doi: 10.1371/journal.pone.0071905 (PMC3754936; doi:10.1371/journal.pone.0071905)
Supplement: Table S1 — Repeated measures T-tests for blood cell types and chemistries. (DOCX) [file pone.0071905.s001.docx]

**Table S1.** Repeated measures T-tests for blood cell types and chemistries.

| **Measurement** | **Admission Mean (SD)** | **24 Hour**  **Mean (SD)** | **Test Result T(p)*** |
| --- | --- | --- | --- |
| WBC | 20.32 (9.45) | 9.03 (2.25) | 5.3309 (0.0001) |
|  |  |  |  |
| HGB | 15.47 (1.25) | 12.51 (1.23) | 11.5398 (<0.0001) |
|  |  |  |  |
| HCT | 46.48 (3.81) | 34.83 (6.82) | 5.6650 (<0.0001) |
|  |  |  |  |
| PLT | 343 (102) | 245 (59) | 6.5625 (<0.0001) |
|  |  |  |  |
| NEU | 77 (8) | 62 (8) | 5.5457 (0.0001) |
|  |  |  |  |
| LYMPH | 13 (5) | 28 (8) | -7.9895 (<0.0001) |
|  |  |  |  |
| NA | 135 (5) | 138 (3) | -2.1884 (0.0448) |
|  |  |  |  |
| K | 5.12 (0.72) | 3.84 (0.45) | 6.3337 (<0.0001) |
|  |  |  |  |
| CO2 | 6 (2) | 25 (3) | -20.0413 (<0.0001) |
|  |  |  |  |
| BUN | 19 (9) | 14 (4) | 3.8579 (0.0015) |
|  |  |  |  |
| CR | 1.33 (0.38) | 0.72 (0.22) | 6.2919 (<0.0001) |
|  |  |  |  |
| BG | 512 (147) | 180 (85) | 8.2570 (<0.0001) |

*Seventeen patients had CBC and BMP collected on admission and 24 hours later. Repeated measures T-test showed statistically significant differences between the admission and 24 hours samples.
