# Supplementary material for: Increased Systemic Th17 Cytokines Are Associated with Diastolic Dysfunction in Children and Adolescents with Diabetic Ketoacidosis
Source: PLoS One. 2013 Aug 27;8(8):e71905. doi: 10.1371/journal.pone.0071905 (PMC3754936; doi:10.1371/journal.pone.0071905)
Supplement: Table S2 — Echocardiography variable comparative analysis for DA (N = 8) and Non-DA (N = 9) groups at T1 (6–12 hrs post admission) or T2 (2–3 weeks/ECHO baseline). (DOCX) [file pone.0071905.s002.docx]

**Table S2.** Echocardiography variable comparative analysis for DA (N=8) and Non-DA (N=9) groups. T1 (6-12 hrs post admission) or T2 (2-3 weeks/ECHO baseline).

| **ECHO Variable** | **Comparison at**  **Echo Time**  **(T1 or T2)** | **DA Group:**  **Mean (SD) or**  **Median(range)** | **Non-DA Group:**  **Mean (SD) or**  **Median(range)** | **Test Result***  **T(p) or**  **Z(p)** |
| --- | --- | --- | --- | --- |
| Mitral Valve E/A Ratio | T2 | 1.96  (0.3862) | 2.33  (0.2397) | 2.4129  (0.0291) |
| Mitral Deceleration Time: HRc (msec) | T1 | 221.375  (10.774) | 188.556  (26.158) | 1.8469  (0.0423) |
| 2D LV Wall thickness: Systole (cm) | T1 | 1.525  (0.1769) | 1.32  (0.2022) | 2.2019  (0.0430) |
| 2D Septum thickness (cm) | T1 | 0.795  (0.7-0.86) | 0.65  (0.6-0.93) | 1.8796  (0.0301) |
| LA Volume (cm^3^) | T1 | 19.5  (5.4511) | 14.111  (4.8848) | 2.1506  (0.0482) |

*Only ECHO measurements with significant differences between DA and Non-DA groups were tabulated.
