# Supplementary material for: Increased Systemic Th17 Cytokines Are Associated with Diastolic Dysfunction in Children and Adolescents with Diabetic Ketoacidosis
Source: PLoS One. 2013 Aug 27;8(8):e71905. doi: 10.1371/journal.pone.0071905 (PMC3754936; doi:10.1371/journal.pone.0071905)
Supplement: Table S3 — Statistically significant Spearman correlations between admission chemistries including BG, HbA1c and cytokines at T1 (6–12 hours) and at T3 (3 months post admission). (DOCX) [file pone.0071905.s003.docx]

**Table S3.** Statistically significant Spearman correlations between admission chemistries including BG, HbA1c and cytokines at T1 (6-12 hours) and at T3 (3 months post admission).

| **Relationship @ Time** | **Spearman Correlation Coefficient** | **p value** |
| --- | --- | --- |
| BUN: IL12p40 @ T1 | r_s_ = 0.5359 | p = 0.0395 |
| BUN: CXCL10 (IP10) @ T1 | r_s_ = 0.6691 | p = 0.0033 |
| BUN: Eotaxin @ T1 | r_s_ = 0.3711 | p = 0.0335 |
| CO2: CX3CL1 (Fractalkine) @ T1 | r_s_ = 0.5730 | p = 0.0203 |
| CO2: MDC @ T1 | r_s_ = 0.5110 | p = 0.0361 |
| CO2: CXCL10 (IP10) @ T1 | r_s_ = 0.3668 | p = 0.0358 |
| CO2: VEGF @ T1 | r_s_ = 0.5418 | p = 0.0247 |
| BG: MCP1 @ T1 | r_s_ = -0.4832 | p = 0.0273 |
| BG: MDC @ T1 | r_s_ = -0.3570 | p = 0.0414 |
| HbA1c: MDC @ T1 | r_s_ = -0.3796 | p = 0.0268 |
| HbA1c: IL1RA @ T3 | r_s_ = 0.7783 | p = 0.0393 |
| HbA1c: IL12p40 @ T3 | r_s_ = 0.6000 | p = 0.0392 |
| HbA1c: MDC @ T3 | r_s_ = -0.4179 | p = 0.0139 |
| BG: IL1RA @ T3 | r_s_ = - 0.7783 | p = 0.0393 |
| BG: GCSF @ T3 | r_s_ = - 0.5328 | p = 0.0336 |
| BUN: GMCSF @ T3 | r_s_ = 0.5552 | p = 0.0256 |

HbA1c had significant (p<0.05) and direct correlations with the cytokines IL-1RA and IL12p40.  Alternatively, BG had significant (p<0.05) but inverse correlations with IL-1RA and GCSF.  The relationships of HbA1c and BG with IL-1RA are strong, accounting for 59% of the variation observed in the pairings.
